# Using gene expression data to identify certain gastro-intestinal diseases

**DOI:** 10.1186/2043-9113-2-20

**Published:** 2012-11-21

**Authors:** Philip S Crooke, John T Tossberg, Sara N Horst, John L Tauscher, Melodie A Henderson, Dawn B Beaulieu, David A Schwartz, Nancy J Olsen, Thomas M Aune

**Affiliations:** 1 Department of Mathematics, Vanderbilt University, Nashville, TN, USA; 2 Research Department, ArthroChip, LLC, Franklin, TN, USA; 3 Department of Medicine, Vanderbilt University School of Medicine, Nashville, TN, USA; 4 Department of Medicine, Penn State Hershey Medical Center, Hershey, PA, USA; 5 Department of Pathology, Microbiology and Immunology, Vanderbilt University School of Medicine, Nashville, TN, USA

## Abstract

**Background:**

Inflammatory bowel diseases, ulcerative colitis and Crohn’s disease are considered to be of autoimmune origin, but the etiology of irritable bowel syndrome remains elusive. Furthermore, classifying patients into irritable bowel syndrome and inflammatory bowel diseases can be difficult without invasive testing and holds important treatment implications. Our aim was to assess the ability of gene expression profiling in blood to differentiate among these subject groups.

**Methods:**

Transcript levels of a total of 45 genes in blood were determined by quantitative real-time polymerase chain reaction (RT-PCR). We applied three separate analytic approaches; one utilized a scoring system derived from combinations of ratios of expression levels of two genes and two different support vector machines.

**Results:**

All methods discriminated different subject cohorts, irritable bowel syndrome from control, inflammatory bowel disease from control, irritable bowel syndrome from inflammatory bowel disease, and ulcerative colitis from Crohn’s disease, with high degrees of sensitivity and specificity.

**Conclusions:**

These results suggest these approaches may provide clinically useful prediction of the presence of these gastro-intestinal diseases and syndromes.

## Background

Inflammatory bowel diseases (IBD), Crohn’s disease (CD), Celiac’s disease (CeD) and ulcerative colitis (UC) are chronic relapsing remitting inflammatory conditions affecting the gastrointestinal tract, primarily the small intestine and colon [1]. CD is most frequently diagnosed in patients in their 20s and UC in their 30s; however, the diagnosis can be made at any age [2]. IBD diagnosis is often straightforward, as disease can be seen by endoscopy or imaging modalities. However, diagnosis can be difficult as patients may experience symptoms consistent with IBD but ultimately have other diagnoses including functional gastrointestinal disorders such as irritable bowel syndrome (IBS) [3-6]. Patients with IBS can have symptoms very similar to those with IBD. IBD can be limited to difficult to evaluate areas of the GI tract such as isolated small bowel disease. Also, within IBD, differentiating between CD and UC can be difficult, especially within patients with severe inflammatory activity, often termed *indeterminate colitis*[7]. When the clinical presentation is severe and an operation including colectomy is indicated, differentiating CD and UC is imperative, as ileal pouch-anal anastomosis (IPAA) is generally contraindicated in CD due to high morbidity [8].

Developing biomarkers that can be easily obtained and allow for the correct diagnosis early into evaluation can avoid costly interventions that expose patients to multiple unnecessary procedures. Blood markers for both IBD and IBS have been sought for decades. For IBD, perinuclear antineutrophil cytoplasmic antibody *(p-ANCA*) and anti-Saccharomyces cerevisiae antibody (*ASCA*) have been reported to be markers for UC and CD, respectively. However, *p-ANCA* is also detected in 10–40% of patients with CD and *ASCA* is detected in 6–14% of patients with UC [1]. Other markers increased in subjects with CD include antibodies to (*a*) *Escherichia coli* outer membrane porin C (Omp-C), (*b*) protein from *Pseudomonas fluorescens*[9] and (*c*) flagellin c-BIR1 (anti-CBIR1) [10], but these markers remain insensitive. In patients with indeterminate colitis, those with one or more positive antibodies, including *ANCA*, *ASCA*, *I2* (antibody to *Pseudomonas fluorescens*), and Omp-C, have significantly higher post-operative complications [11]. Other inflammatory biomarkers such as C-reactive protein, fecal calprotectin, and fecal lactoferrin differentiate IBD from other gastrointestinal disorders such as IBS [5], but tests do not differentiate among various types of inflammatory colitides [12].

Additional biomarker candidates include DNA variants, differences in RNA transcript abundances, including mRNAs, microRNAs, long intergenic non-coding RNAs, proteins, or metabolites. A general view is that different profiles of biomarkers could provide useful information to guide clinical decision-making; from diagnosis to choice of optimal therapies and in some cases these biomarker profiles are being implemented in clinical practice [3,12-24]. Searches for optimal biomarker profiles can be achieved using clustering methods *e.g*., heirarchical clustering, K-means clustering, which depend upon the general ability to find common features across a sample population or forms of linear discriminate analysis, which depend upon the ability to find linear combinations of features that have the ability to separate two or more classes. The former method is a common method to analyze large numbers of features, such as microarray data whereas the latter is a more common method for analysis of smaller numbers of features. Both methods are suitable for further analyses using machine learning methods such as support vector machines, logistic regression, principal components analysis or prediction analysis for microarrays. Using a form of linear discriminant analysis, we have attempted to employ mRNA transcript profiles to distinguish between subjects with multiple sclerosis and other comparator groups [25,26]. Our results clearly demonstrate that mRNA transcript profiling has the capacity to distinguish between MS, even early in the disease process, and homogeneous comparator groups, such as healthy subjects (CTRL), or subjects with clinically related diseases such as neuromyelitis optica or transverse myelitis. Thus, these binary comparisons can produce a test of exclusion of multiple sclerosis. Here, we applied this approach to IBD and IBS. Our results demonstrate that distinct mRNA profiles accurately discriminate IBD from CTRL, IBS from CTRL, IBD from IBS, and CD from UC with high degrees of sensitivity and specificity. We propose these approaches may provide useful guides for clinical decision-making.

## Methods

### Human subjects

Blood samples collected in PAXgene tubes were obtained from CTRL, IBS, CeD, CD or UC subjects. Diagnosis of IBD, both CD and UC, was made by colonoscopy or sigmoidoscopy and tissue biopsy to localize inflammation to all layers of the intestinal wall (CD) or only the inner lining layer (UC). Diagnosis of IBS was made by the absence of pathologic damage in the colon after examination by colonoscopy or sigmoidoscopy. Inclusion criteria were diagnosis by a gastro-intestinal specialist using these methods. Age, race and gender were not statistically different among the different study groups. Time of blood draw, for example, morning/afternoon clinics, was also not statistically significant among the different study groups. Relevant institutional review board (Vanderbilt University School of Medicine and University of Texas Southwestern Medical School) approval was obtained from all participating sites. Informed consent was obtained from all subjects.

### MRNA transcript determination

Total RNA was purified using Qiagen’s RNA isolation kits using standard protocols and was reverse-transcribed using poly-A primers uisng Superscript III (Invitrogen, Carlsbad, CA, USA). A TaqMan Low Density Array (TLDA) was designed to analyze expression levels of 44 target genes and of four *housekeeping genes* in 300 ng cDNA. The gene probes on the TLDA plate were: *ABR, ACTB, ACTR1A, ADAMTSL4, ANAPC1, APOBEC3F, ASL, B2M, BRCA1, CD55, CDH1, CDKN1B, CHEK2, CSF3R, CTSS, EPHX2, EXT2, FOS, FOSL1, GAPDH, GATA3, GNB5-1, GNB5-1, GSTM4, HLA-DRA, HRAS, IFI27, IL11RA, JUN, KRAS, LEPREL4, LLGL2, NRAS, OAS1, ORC1L, PGK1, PMAIP1, POU6F1, RANGAP1, SC65, SPIB, TAF11, TBP, TGFBR2, TP53-1 TP53-2, TXK*. *GNB5*-1 and −2 and *TP53*-1 and −2 interrogate different exon-intron junctions [26]. Inclusion of the specific gene targets was based upon the following criteria: (*a*) previous studies demonstrating differential expression among control and multiple autoimmune diseases, (*b*) protein products possess known inflammatory functions, (*c*) expression levels change in response to pro-inflammatory stimuli (cytokines), and/or (*d*) protein products have known roles in cell cycle progression and/or apoptosis. Patient diagnosis was blinded for all experimental procedures. Relative expression levels were determined directly from the observed threshold cycle (*C*_*T*_).

### Ratioscore and support vector machine (SVM) methods

Principal Component Analysis (PCA) was applied directly to the normalized gene expression data using MATLAB’s *Bioinformatics Toolkit* (The MathWorks, Inc.) and other techniques to identify a lower dimensional space of gene expressions that could be used to classify controls from cases. The results were disappointing and we concluded that looking at ratios of the gene expression data may be a more productive approach. The computational algorithm and permutation testing strategy employed to identify discriminatory combinations of ratios to create the *ratioscore* (our terminology) have been previously described [26]. For completeness, we summarize the algorithm used in the Ratioscore Method below. Let *D* denote the set of gene-expression levels associated with the disease group and *C* denote the set of gene-expression levels associated with the control group. The algorithm searches for the “best” set of gene ratios that partitions *D* and *C*:

• 80% of the control and disease groups are randomly selected. Gene-expression level ratios are formed for elements in *D* and *C*. For each ratio, the number of elements in the disease group that are larger than the largest ratio in the control group is computed. The top 500 ratios that separate elements in *D* and *C* are saved. This calculation is repeated 200 times resulting in a set of 200 subsets of ratios (each subset having 500 ratios).

• The 500 subsets are then processed looking for the smallest number of ratios, *R*={*r*_1_, *r*_2_, …, *r*_n_}, that produce the maximum of separation of *D* and *C*. Associate with each of the ratios in *R*, there are threshold values, *T*={*t*_1_, *t*_2_, …, *t*_n_}, which correspond to the highest value in the control group for each of the ratios in *R*.

• For each member of the disease group *D*, the ratios in *R* are computed, {*α*_1_, *α*_2_, …, *α*_n_}. If *α*_*i*_ ≥ *t*_*i*_, then we assign the ratio a 1; otherwise, it is assigned a 0. In this way, we generate an *n*-tuple of 1’s and 0’s for each member of *D*. For example, if *n* = 6, then a typical 6-tuple would be {1,1,0,0,1,0}. This would mean that this individual in the disease group would have 3 ratios that exceed the corresponding ratios in the control group.

• Lastly, the percentage of members in the disease group that have nonzero *n*-tuples is calculated. The larger the percentage, the better the separation of *D* and *C*.

The algorithm alllows one to identify the smallest number of ratios that partitions the case and control groups.

Two support vector machines (SVM) were independently created and trained using ratios identified by the Ratioscore Method. The first SVM was coded in *Mathematica* (Wolfram Research, Inc*.*) and the second SVM employed *LS-SVMLab* software (http://www.esat.kuleuven.be/sista/lssvmab). We decided to use the two independently developed SVM since the choice of kernels, optimization algorithms, and the training algorithms can produce differing results. There was little difference in the performance of the two machines when classifying the different case–control combinations. To confirm the results of the Ratioscore Method and the SVM approaches, logistic regression was employed to separate to the case and control sets using the gene ratios. Its performance was in line with the other two approaches and hence, we have chosen not to report these results.

### Statistical analysis

The Welch’s-corrected T-test not assuming equal variances was employed to calculate p-values in two-way comparisons. Fisher’s exact test was employed to calculate p-values in 2 by 2 comparisons. The Bonferroni’s method was employed to correct for multiple testing [27].

## Results

### Gene-expression patterns in distinct gastrointestinal diseases

**Figure 1:**
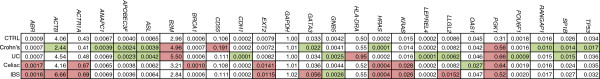
**Gene-expression profiles in multiple gastrointestinal disorders**. Expression levels of 44 target genes were determined by quantitative RT-PCR and normalized to expression of *GAPDH*. Expression levels of 25 genes are shown; expression levels of the remainder were not statistically different between CTRL and any disease cohort. Results are expressed as transcript levels of individual genes relative to transcript levels of *GAPDH* using the formula: 2^(GAPDH *C*^_*T*_^-target gene *C*^_*T*_^*)*^. Genes are identified that showed statistically significant (p-value < 0.05 after Bonferroni’s correction) increased (red boxes) or decreased (green boxes) expression in individual disease cohorts relative to CTRL subjects.

CTRL, IBD (CD and UC), IBS subjects were recruited from multiple sites within the United States. Demographic characteristics of the different gastrointestinal disease cohorts were not statistically different from the CTRL cohort (Table 1). We measured expression patterns of a common set of genes assayed using a common platform in CTRL and subjects with different gastrointestinal conditions, CD and UC, IBS, and CeD. Genes for analysis were selected from prior microarray studies [20,26]. Gene transcript levels were determined by quantitative RT-PCR and normalized to *GAPDH* transcript levels. We employed a heatmap to depict those genes differentially expressed in individual subject cohorts relative to the CTRL cohort, p-value < 0.05 (after Bonferroni correction for multiple testing; see Figure 1 with red = over-expressed gene, green = under-expressed gene). Ratios of transcript levels of individual genes in the indicated disease cohorts relative to *GAPDH* were calculated and depicted within each box. Each disease exhibited an underlying unique pattern of gene-expression. However, these profiles were sufficiently overlapping to prohibit accurate discrimination of one disease from another disease using the expression profile alone. For example, while *PGK1* was over-expressed in all four conditions, *ABR*, *ACTR1A*, *EXT2*, *HRAS*, and *KRAS* were over-expressed in CeD and IBS but not CD and UC. Similarly, *APOBEC3F*, *ASL*, and *SPIB* were under-expressed in CD and UC, but not CeD and IBS. Other genes, *ANAPC1*, *RANGAP1*, and *TP53*, were only under-expressed in CD. Certain genes, *e.g*., *APOBEC3F*, *ASL*, *GNB5*, *SPIB*, were only under-expressed relative to the CTRL cohort, while other genes, *e.g*., *ACTB*, *GATA3*, *HRAS*, and *LLGL2*, were under-expressed in specific disease cohorts relative to CTRL but over-expressed in other disease cohorts relative to CTRL. Thus, each gene was differentially expressed in at least one disease cohort relative to CTRL. However, each individual disease cohort did not possess a unique expression profile distinguishing it from all other disease cohorts. For these reasons, we decided to look at other separation techniques.

**Table 1:** Demographic characteristics of the different subject populations

	***#***	**AGE yrs**	P *	**GENDER (% F)**	***P***	**ETHNICITY (% C/AA/As/H)**	***P***
IBD	97	40±9	NS	62	NS	92/5/0/1	NS
CD	46	38±10	NS	63	NS	91/4/0/0	NS
UC	40	41±8	NS	59	NS	93/5/0/2	NS
IBS	44	43±10	NS	79	NS	90/7/0/3	NS
CeD	16	44±12	NS	69	NS	100/0/0/0	NS
CTRL	113	41±11		67		89/9/0/2	

*P calculated by Student T-test (Age) or Fisher’s exact test, NS: *p-value* > 0.05.

^†^C, Caucasian; AA, African American; As, Asian; H, Hispanic.

### Discrimination of IBD or IBS from CTRL based upon gene-expression ratios

Initially, we employed standard methods of microarray analyses including unsupervised heirarchical clustering, supervised heirarchical clustering, and principal components analysis using the TIGR microarray software Multiexperiment Viewer to segregate patient groups. After normalization to *GAPDH*, gene expression data from IBD samples or IBS samples and CTRL samples were analyzed using unsupervised and supervised heirarchical clustering using all genes or only those genes whose expression was statistically significant using the supervised T-test. We found that unsupervised heirarchical clustering segregated 72% of IBD samples in one major branch and 28% of IBD samples in the second major branch. Similarly, 36% of CTRL samples were segregated into the branch with most of the IBD samples while 64% of CTRL samples were segregated into the alternate branch. Comparison of IBS and CTRL using unsupervised heirarchical clustering also did not produce the desired level of discrimination between case and control cohorts. Supervised heirarchical clustering and principal components analysis produced a similar low level of overall accuracy.

For these reasons, we turned to a type of linear discriminant analysis classifier (Ratioscore Method) that we employed previously to discriminate subjects with multiple sclerosis from different control cohorts. We employed a search algorithm to identify those ratios of gene-expression levels in which the greatest number of subjects in the test group possessed a ratio value greater than the highest ratio value in the comparator group. We employed a second algorithm to perform permutation testing of one subject group to identify the optimum set of discriminatory ratios. CeD was excluded from this analysis due to the low number of cases in this cohort. Examination of expression levels of ratios of genes rather than individual genes offered the following advantages. First, ratios normalized for differences in mRNA or cDNA template quantity and quality among different samples. Second, ratios obviated the need for inclusion of a *housekeeping genes* in the analysis and the assumption that expression levels of *housekeeping genes* did not vary among different subject populations. Third, comparisons of ratios or combinations of ratios may more accurately identify cellular phenotypes that may contribute to disease. For example, a ratio containing one gene in the numerator that is over-expressed in the case cohort relative to the control cohort and one gene in the denominator that is under-expressed in the case cohort relative to the control cohort should produce a greater ratio value difference between individuals in the two cohorts than a single expression value. Fourth, *ANAPC1*, *RANGAP1*, and *LEPREL4* genes encode unique proteins and each participates in mitosis [28-33]. Thus, a defect in expression of any one of these genes could produce a common cellular phenotype; a defect in mitosis, and for example, one subject with a given disease may exhibit a deficiency in expression of *ANAPC1* while a second individual with the same disease may exhibit a deficiency in expression of *RANGAP1* and a third with the same disease may exhibit a defect in *LEPREL4* expression levels. Any of these defects has the potential to produce a common cellular phenotype. Our approach makes it possible to capture each subject as positive for a given disease. We refer to this as the Ratioscore Method.

**Figure 2:**
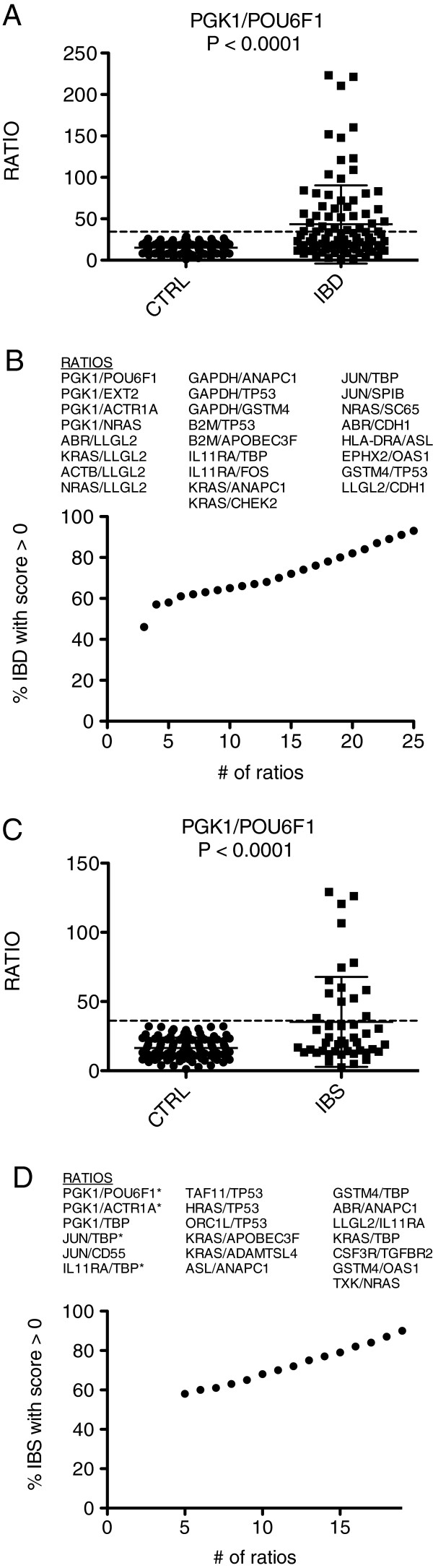
**Discrimination of IBD from CTRL and IBS from CTRL using the ratioscore system.** (**A**) Ability of a single ratio, *PGK1/POU6F1*, to discriminate IBD and CTRL subjects. (**B**) The most discriminatory 25 gene-expression ratios were identified to segregate IBD and CTRL subjects. The ratioscore method was applied to combine ratio performance into a single discriminator. (**C**) Ability of a single ratio, *PGK1/POU6F1*, to discriminate IBS and CTRL subjects. (***D***) The most discriminatory 19 gene-expression ratios were identified to segregate IBS and CTRL subjects. The ratioscore method was applied to combine ratio performance into a single discriminator * indicates ratios found in both IBD:CTRL and IBS:CTRL comparisons.

We applied this approach to determine how accurately it would distinguish subjects with IBD or IBS from CTRL. First, we identified ratios capable of discriminating IBD subjects from CTRL. Second, we applied a re-sampling permutation testing strategy to identify ratios that consistently displayed high discriminatory power. Third, we identified the smallest number of ratios producing the greatest discrimination between two comparator groups. The single ratio with the greatest discriminatory power was *PGK1/POU6F1* (Figure 2A). Using this ratio, 30% of IBD subjects achieved a ratioscore value higher than all CTRL subjects and were awarded one point. A combination of 25 ratios produced a scoring panel where 100% of CTRL subjects achieved a score of 0 and 94% of IBD subjects achieved a ratio ≥ 1 (Figure 2B). Thus, we conclude that gene-expression ratios we identified accurately distinguished IBD subjects from CTRL.

We continued our analysis to determine how well IBS and CTRL cohorts were differentiated. Interestingly, the optimum ratio that distinguished the IBD cohort from the CTRL cohort, *PGK1*/*POU6F1*, was also the optimum ratio that distinguished the IBS cohort from the CTRL cohort (Figure 2C). We identified a total of 19 ratios that, in combination, produced a point system whereby 100% of CTRL subjects achieved a score of 0 and 90% of IBS subjects achieved a ratio ≥ 1 (Figure 2D). Thus, even though IBS is generally considered not to be an inflammatory disease, we conclude our approach accurately distinguishes these subjects from the CTRL group.

### IBS-IBD discrimination based upon the Ratioscore Method

**Figure 3:**
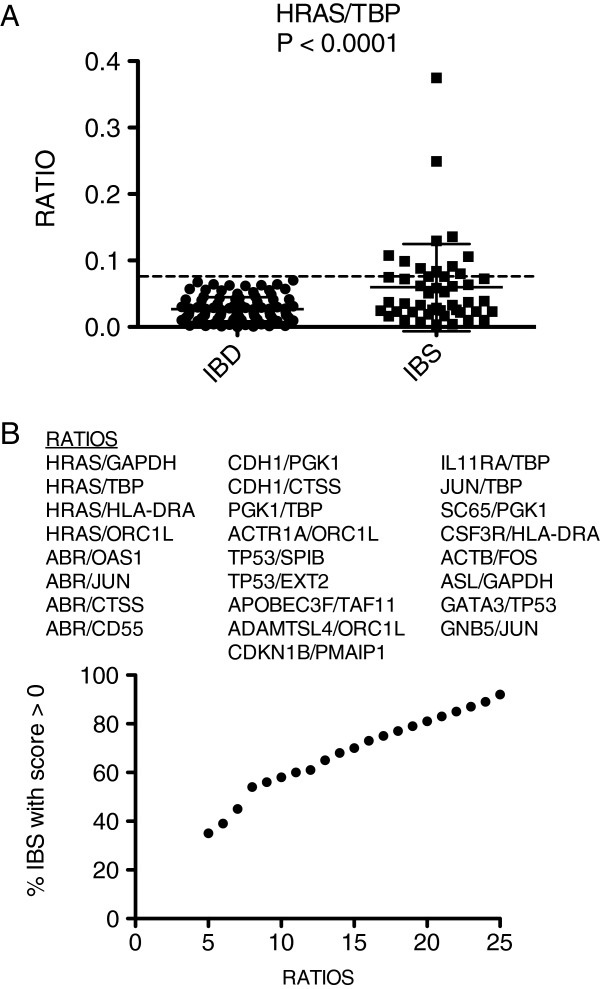
**Discrimination of IBD from IBS using the Ratioscore system.** (**A**) Ability of a single ratio, *HRAS/TBP*, to discriminate IBD and IBS subjects. (**B**) The most discriminatory 25 gene-expression ratios were identified to segregate IBD and IBS subjects. The ratioscore system was applied to combine ratio performance into a single discriminator.

Next, we assessed our ability to distinguish IBS and IBD cohorts. The optimum ratio we identified was *HRAS/TBP*, p-value < 0.0001 (Figure 3A). We identified a total of 25 ratios that, combined, produced a ratioscore whereby 100% of IBD subjects achieved a score of 0 and 92% of IBS subjects were awarded a ratio ≥ 1 (Figure 3B). Thus, we conclude that the ratioscore method was capable of discriminating between subjects with IBD and subjects with IBS.

### UC-CD discrimination disease based upon the Ratioscore Method

**Figure 4:**
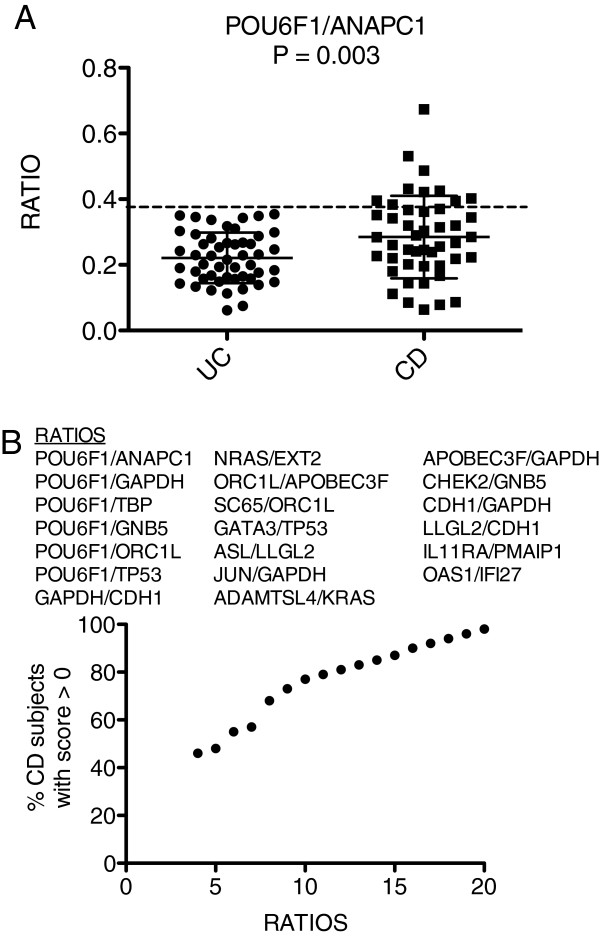
**Discrimination of UC from CD using the Ratioscore system.** (**A**) Ability of a single ratio, *POU6F1/ANAPC1*, to discriminate UC and CD subjects. (**B**) The most discriminatory 20 gene-expression ratios were identified to segregate UC and CD subjects. The Ratioscore System was applied to combine ratio performance into a single discriminator.

Finally, we determined if our approach accurately discriminated between the two inflammatory bowel diseases, UC and CD. The optimum ratio was *POU6F1*/*ANAPC1*, p-value = 0.003 (Figure 4A). We identified a total of 20 ratios that, in combination, produced a point system that awarded 100% of UC subjects a score of 0 and 98% of subjects with CD a ratio ≥ 1 (Figure 4B). Thus, the Ratioscore Method accurately discriminated between the two major subclasses: IBD:UC and IBD:CD.

### Disease discrimination based upon the SVM method

Support Vector Machines (SVM) were also employed to classify the data into two distinct groups. The inputs for the SVM were the same ratios used to calculate the ratioscores. For example, when separating IBS patients from CTRL subjects, the same 19 ratios of normalized gene-expression ratios employed to compute the ratioscore were used as input to the SVM. In the SVM calculations, we chose the radial basis kernel (RBK) to perform the *kernel trick*. This kernel contains a fitting parameter *β*. We also used the “soft margin” approach to the fitting of the hyper-surface that separates the two groups (cases and controls). This introduced a second fitting parameter *C*. Programs written in *Mathematica* (Wolfram Research, Inc.) were created and random training subsets of the two groups were chosen to find the parameters, *β* and *C*. Each training subset consisted of 60% of the total dataset. The values of the two fitting parameters that produced the smallest number of incorrect cases and controls were used to define the SVM. This SVM analysis also accurately discriminated the different subject groups: (*i*) IBD and CTRL, (*ii*) IBS and CTRL, (*iii*) IBD and IBS, and (*iv*) CD and UC (Table 2).

**Table 2:** Case/control discrimination by support vector machines (SVM #1)

			**Case**		**CTRL**	
**Comparison**	**Total #**	**Training set % of total**	**TP #**	**FN #**	**TN #**	**FP #**
IBD* vs. CTRL	209	60	95	1	100	13
IBD* vs. CTRL	160	60	47	0	96	17
IBD* vs. IBS	143	60	45	2	86	10
CD* vs. UC	85	60	45	2	31	7

*Case cohort.

^†^TP = true positive, FN = false negative, TN = true negative, FP = false positive.

A second SVM was also employed using *LS-SVMLab* software (http://www.esat.kuleuven.ac.be/sista/lssvmlab) to validate the SVM created with *Mathematica*. The procedure for training the SVM followed the following algorithm:

• *X* (*X* = 50%, 60%, and 80%) was randomly selected from the total set of data and used to train the SVM.

• On the selected training set, *L-fold cross-validation* was performed. In this type of training a certain fraction of the training set was omitted from training and the remaining portion of the partial training set was used to estimate the parameters of the SVM. This was repeated *L* times. We used *L* = 10. At the completion of the training, a composite estimate for the parameters was obtained.

• Once the SVM was trained on *X*% of the total data, the SVM was applied to the total data set.

Numbers of correct and incorrect classifications were tabulated for total sets (training and validation), training sets and validation sets (Table 3). Overall accuracy in the training sets was greater than overall accuracy of the validation sets. The different training sessions did not produce much variation in the overall accuracy of the corresponding validation sets. Using the above algorithm, two different kernels, a polynomial kernel and Radial Basis Function (RBF) kernel, were used to create different machines. Overall, the SVM with the RBF kernels performed somewhat better than the polynomial kernels.

**Table 3:** Overall accuracy in total, training and validation sets by SVM #2 method

**TOTAL SET**				**TRAINING SET**				**VALIDATION SET**			
Tc*	**Ti**^**†**^	**TOTAL**^**‡**^	**%I**^**§**^	**Tc**	**Ti**	**TOTAL**	**%I**	**Tc**	**Ti**	**TOTAL**	**%I**
80% IBS-C (RBF kernel)											
152	8	160	5	124	3	127	2	28	4	33	12
80% IBD-C (RBF kernel)											
207	2	209	1	160	0	166	0	41	2	43	4
80% IBD-IBS (RBF kernel)											
139	4	143	3	111	1	113	1	27	3	30	10
60% CD-UC (RBF kernel)											
77	7	85	9	47	4	51	8	31	3	34	11
60% IBS-C (polynomial)											
150	10	160	6	91	4	95	4	59	6	65	9
60% IBD-C (polynomial)											
195	14	209	7	88	7	95	7	107	7	114	6
60% IBD-IBS (polynomial)											
124	19	143	13	78	8	85	8	46	11	58	19
60% CD-UC (polynomial)											
76	9	85	10	47	4	50	8	30	5	35	14

*Tc, total number correct in designated set.

^†^Ti, total number incorrect in designated set.

^‡^Total, total number of cases and controls analyzed in designated set.

^§^% I, incorrect percentage of case:control calls in designated set.

**Figure 5:**
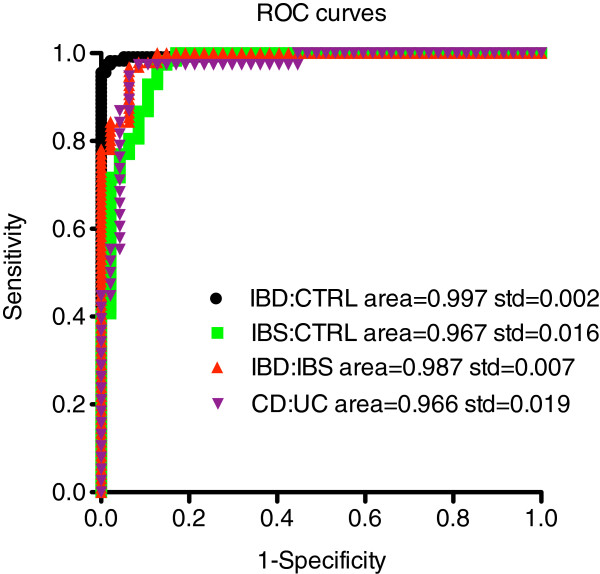
**ROC curves derived from SVM #2 method.** Sensitivity, specificity, and AUC were determined using the Mathematica program for the following comparisons: IBD:CTRL, IBS:CTRL, IBD:IBS, and CD:UC.

**Figure 6:**
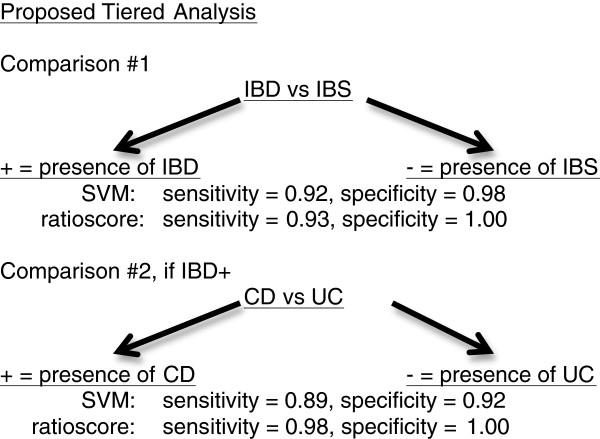
Proposed tiered analyses to discriminate subjects with IBD or IBS and, if positive for IBD, to discriminate between CD and UC.

This second SVM was used to discriminate between the different subject groups, IBD and CTRL, IBS and CTRL, IBD and IBS, and CD and UC producing levels of sensitivity and specificity comparable to the Ratioscore Method or the first SVM method (Table 4). We determined receiver operating characteristic (ROC) curves from data produced by the second SVM method. The area-under-the-curve (AUC) for each comparison exceeded 0.96 (Figure 5). The IBD:CTRL comparison produced the greatest overall accuracy (AUC of 0.997). Thus, a tiered approach, using either ratioscore or SVM analysis, can be employed to segregate between IBD and IBS, first, followed by segregation between CD and UC if a subject is IBD positive. This approach produced high levels of sensitivity and specificity at both tiers of the analysis (Figure 6).

**Table 4:** Sensitivity and specificity produced by Ratioscore and two SVM methods

**Method**	**Ratioscore**		SVM #1*		SVM #2*	
	**Sensitivity**	**Specificity**	**Sensitivity**	**Specificity**	**Sensitivity**	**Specificity**
IBD vs. CTRL	0.94	1.00	0.97	0.94	0.99	0.97
IBS vs. CTRL	0.91	1.00	1.00	0.68	0.85	0.99
IBD vs. IBS	0.93	1.00	0.97	0.91	0.92	0.98
CD vs. UC	0.98	1.00	0.94	0.85	0.89	0.92

*Training set = 80% of total, **Training set = 60% of total.

Sensitivity = # true positives/(# true positives + # false negatives).

Specificity = # true negatives/(# true negatives + # false positives).

In the above discussion, two support vector machines were independently created and trained using the ratios identified by the Ratioscore Method. There was little difference in the performance of the two machines when used to classify the different case–control combinations. One advantage of the SVM-based approach is that it can be used to classify more than two groups. As an example of classification into three groups, we considered data for UC (N = 40), CD (N = 46), and CTRL (N = 113). Using gene ratios determined by comparing CTRL (controls) to UC+CD (cases), the SVM identified 99.8% of CTRL, 72.5% of UC, and 56.5% of the CD. Hence, the performance of the tertiary classification was not as accurate as the binary classifications. However, the tertiary classification was improved by using a different set of gene ratios, *e.g*., the union of the set from CTRL vs. CD, CTRL vs. UC, and CD vs. UC. In this case, the SVM identified 99.1% of CTRL, 100% of UC, and 84.8% of CD. One factor that may contribute to this increased accuracy is that the number of gene ratios used in the training of the SVM was increased from 23 ratios to 49 thus introducing additional parameters into the SVM structure.

## Discussion

**Figure 7:**
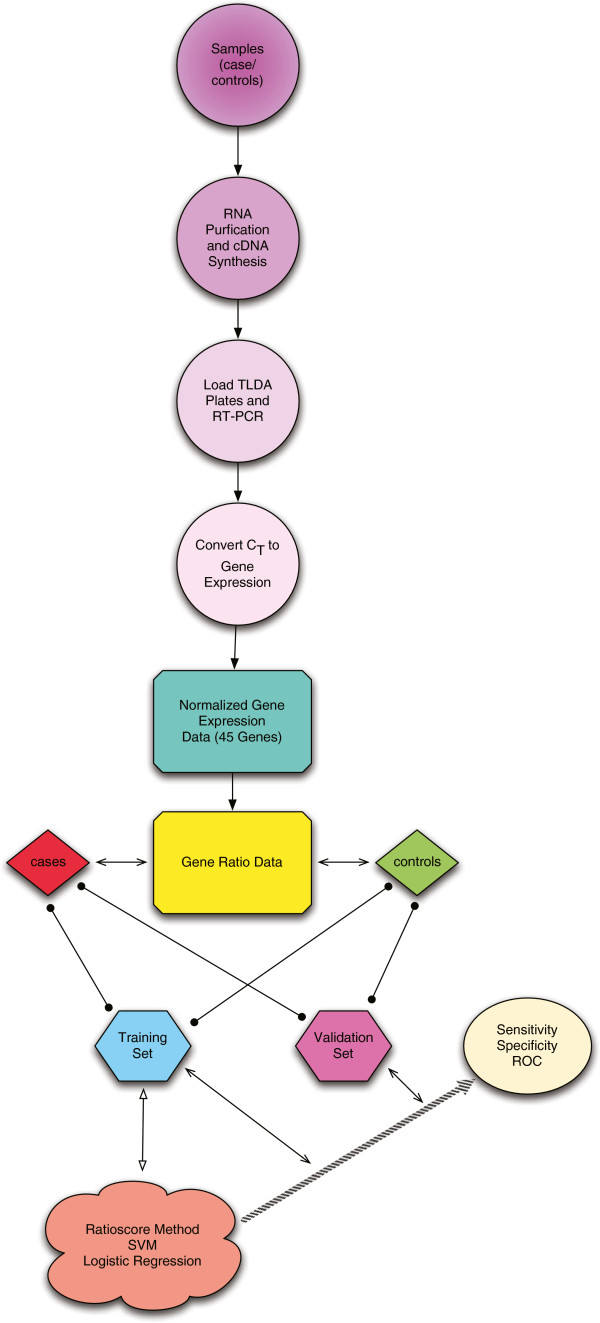
A flow chart of the processing of the data and creation of the classifiers.

IBS and IBD can exhibit overlapping clinical symptoms making diagnosis difficult without invasive procedures [4,12,34]. Therapy and medication for IBS and IBD are vastly different and incorrect diagnosis and treatment plans have significant consequences. Differentiation between UC and CD can also be difficult, having important implications when considering medical and operative treatment options. For example, *ASCA* and *p-ANCA* have clinical utility in diagnosing IBD. *ASCA IgA* is found in 35-50% of patients with CD but < 1% of patients with UC. *ASCA IgG* is found in 50-80% of patients with CD but only 20% of patients with UC. In contrast, atypical *p-ANCA* is found in 70% of UC patients but only 20% of CD patients [19]. Here, we describe a relatively non-invasive procedure capable of accurately discriminating between (*a*) IBS and IBD, and (*b*) the two forms of IBD, UC and CD, using three independent methods based upon transcript levels in blood of a discrete set of genes. Each method employs the same input, which are multiple ratios of expression levels of two genes. The analytic methods, ratioscore, two SVM methods, and logistic regression, produce similar levels of overall accuracy determined by ROC curves which exceed 95%. We have summarized the overall process of going from the raw samples to classification in Figure 7.

In contrast, biomarkers for IBS are non-existent and diagnosis largely depends upon the absence of pathological findings in the colon. Previously identified experimental biomarkers to distinguish UC and CD clearly do not perform with the same degree of accuracy as experimental approaches described here. Thus, we propose these gene expression ratio tests using the Ratioscore Method, SVM, or logistic regression for analysis represent simple non-invasive tests that could accurately classify patients to IBS or IBD categories and IBD patients to UC or CD categories even without colonoscopy or sigmoidoscopy and tissue biopsy.

UC and CD are chronic inflammatory autoimmune diseases. Using various strategies, numerous studies have identified unique gene-expression signatures in blood or peripheral blood mononuclear cells (PBMC) associated with different autoimmune diseases [22]. Some are unique to a single autoimmune disease, some discriminate between two autoimmune diseases and some are shared among multiple autoimmune diseases. Thus perhaps it is not too surprising that we could employ a similar strategy to identify gene-expression signatures capable of discriminating the two forms of IBD, UC and CD, or IBD from CTRL or IBD from IBS. Somewhat surprising is that IBS can be readily distinguished from CTRL. IBS is a disorder whose etiology and pathogenic mechanisms are incompletely understood [4]. Our results clearly demonstrate that IBS possesses an underlying gene-expression signature. One possibility is that IBS possesses an unrecognized mucosal pathology sensed by the immune system and expressed by changes in transcript levels of specific genes. Another possibility is that IBS generates expression of cytokines, chemokines, adhesion molecules, neurotransmitters or other mediators read by the immune system. In support of this notion, over-expression of *PGK1* is associated with IBS, CeD, CD, and UC and *PGK1* is known to be induced by hypoxia and may be induced by other forms of stress, inflammation or generalized mucosal irritation [35]. Further, *ABR*, *ACTR1A*, *EXT2*, *HRAS*, and *KRAS* are over-expressed in both IBS and CeD but not CD and UC. In contrast, *APOBEC3F*, *ASL* and *SPIB* are under-expressed in CD and UC, but not IBS and CeD. Thus, the IBS gene-expression signature is more similar to the CeD gene-expression signature and the UC signature is more similar to the CD signature. It is uncertain if this suggests that IBS may bear additional relationships to CeD. An improved understanding of mechanisms producing differences in levels of specific gene transcripts in IBS may further our understanding of the pathogenesis of IBS.

## Conclusions

Limitations to our study include selection of patients with pre-existing diagnoses of IBS and IBD, as this may not completely represent patients in the general population in whom these tests may be performed. However, in other studies we have shown that subjects with clinically isolated syndrome, a precursor of multiple sclerosis, who progress to a diagnosis of multiple sclerosis score positive in ratioscore- or SVM-based analyses, similar to those described here. This may suggest that subjects with initial clinical symptoms associated with IBD or IBS, CD or UC, may be discriminated by this approach. Future longitudinal approaches are planned to evaluate utility of these tests. Additional methods, such as analysis of gene-expression ratios in multi-dimensional space rather than binary space may improve the diagnostic capabilities of these tests. We employed three independent approaches to evaluate the ability of gene-expression ratios to discriminate subjects with gastro-intestinal diseases with overlapping clinical symptoms and each produced high degrees of specificity and sensitivity. Thus, these minimally invasive tests may assist in excluding or establishing a diagnosis of IBS or IBD, CD or UC.

## Abbreviations

IBD: Inflammatory bowl disease; IBS: Irritable bowel syndrome; CD: Crohn’s disease; UC: Ulcerative colitis; CeD: Celiac’s disease; CTRL: ConTRoL (healthy subjects); PBMC: Peripheral blood mononuclear cell; SVM: Support vector machine; RBF: Radial basis function; RBK: Radial basis kernel.

## Competing interests

NJO and TMA are co-owners of ArthroChip, LLC. No conflicts exist for other authors.

## Authors’ contributions

SNH, DAS and DBB were responsible for the collection and cataloging of the patient data. JTT and MAH performed the processing of the patient data. PSC, JLT, NJO and TMA developed the modeling and computational processing of the laboratory data.
